# Choroidal neovascularization associated with coloboma of the choroid: A series of three cases

**DOI:** 10.4103/0301-4738.77043

**Published:** 2011

**Authors:** Muna Bhende, G Suganeswari, Lingam Gopal, Pramod S Bhende, Lekha Gopal, Chetan Rao

**Affiliations:** Shri Bhagawan Mahavir Vitreoretinal Service, Sankara Nethralaya, 18 College Road, Chennai - 600 006, India

**Keywords:** Anti-VEGF therapy, bevacizumab, choroidal coloboma, choroidal neovascular membrane, photodynamic therapy

## Abstract

Choroidal neovascularization (CNV) is a rare complication associated with coloboma of the choroid. We describe three cases of coloboma choroid where there was loss of vision due to CNV development at the edge of the coloboma. One was managed by photodynamic therapy alone and two were managed by a combination of reduced fluence PDT and intravitreal bevacizumab. Significantly we noted that one treatment session was sufficient to achieve regression of the CNV and improvement in visual acuity.

Choroidal coloboma is a developmental anomaly occurring from incomplete embryonic fissure closure. Associated findings include microphthalmia, cataract, retinal detachment and rarely choroidal neovascularization (CNV). We report three cases of CNV arising from the edge of the coloboma which responded to a single treatment session of photodynamic therapy (PDT) alone (Case 1) or PDT combined with intravitreal bevacizumab (Cases 2 and 3).

## Case Reports

### Case 1

**Figure 1:**
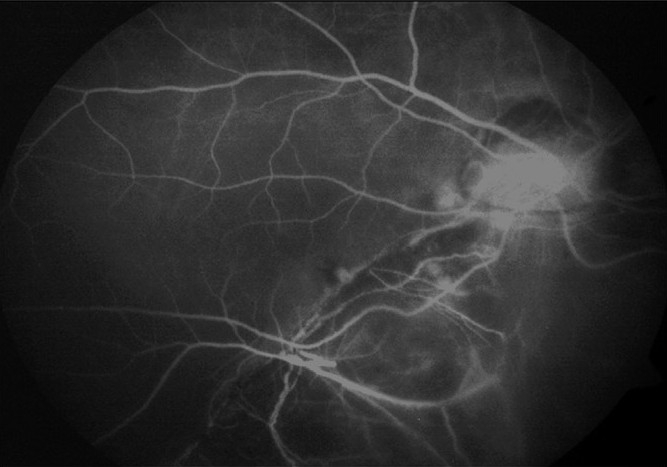
Pretreatment FFA of Case 1, showing a small CNV at the edge of the coloboma

**Figure 2:**
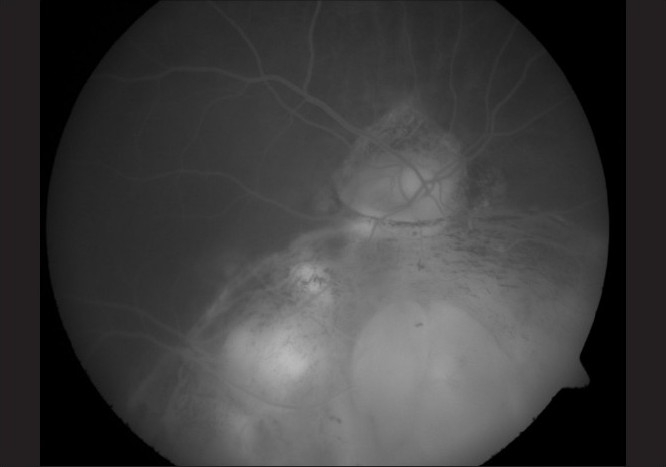
Post-treatment FFA of Case 1, showing decrease in intensity of leakage and RPE atrophy following PDT

A 56-year-old female presented with recent difficulty in reading with the right eye (RE). Past history was significant for the presence of choroidal colobomas in both eyes, cataract surgery in both eyes, intraocular lens (IOL) implantation and scleral buckling in the left eye (LE). She was using timolol maleate 0.5% drops in both eyes. She gave history of coronary artery disease and angioplasty for the same. On examination, best corrected visual acuity (BCVA) in RE was 20/63 N8 and 20/1200 N18 in LE. RE was aphakic with a patent superior iridotomy and an inferior iris coloboma. There was significant posterior capsular opacification. Fundus view was hazy and showed an attached retina with a choroidal coloboma just reaching the macula. LE was pseudophakic with a superior patent peripheral iridotomy and a poorly dilating pupil. Fundus examination showed an attached retina with a coloboma of the choroid and a peripheral buckle effect. Intraocular pressures were 18 and 16 mm Hg. The patient underwent YAG laser capsulotomy in RE. Following this, BCVA was 20/63 N6. The foveal area showed a localized subretinal hemorrhage and grayish subretinal lesion. Fundus fluorescein angiography (FFA) (Ziess FF 450 with Visupac3.2.1) and optical coherence tomography (Stratus OCT version 5.0.1) were performed. A juxtafoveal classic CNV [Fig. 1] was diagnosed on FFA. The patient underwent uneventful standard fluence PDT using verteporfin dye and spot size of 1.64 mm. Subsequent visits showed regression of the CNV on FFA and OCT with transmission defects on FFA due to retinal pigment epithelial atrophy corresponding to the PDT spot [Fig. 2]. When last examined 39 months after treatment, BCVA was 20/32 N6 in RE with fundus showing mild pigmentary changes at the fovea.

### Case 2

**Figure 3:**
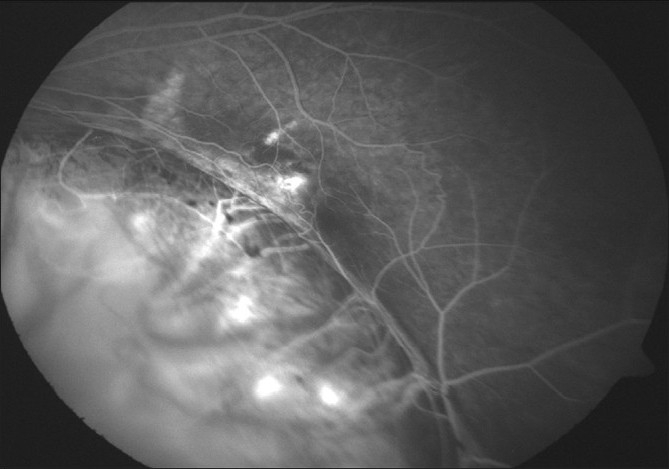
Pretreatment FFA of Case 2, showing a CNV at the edge of the coloboma

**Figure 4:**
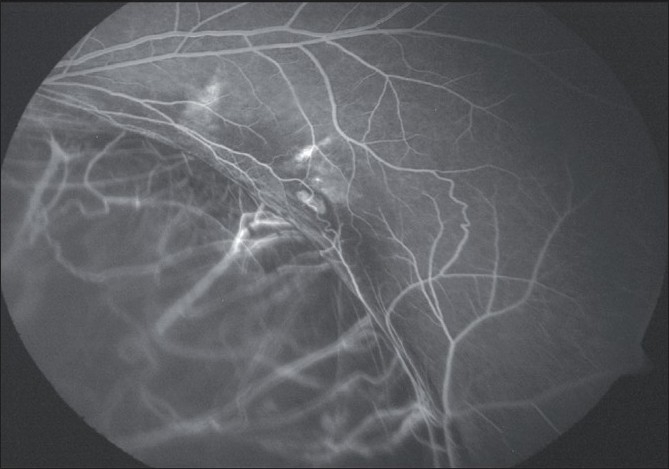
Post-treatment FFA of Case2, showing regression of the CNV after PDT and bevacizumab

A 21-year-old female was evaluated for recent onset of blurred vision in LE. She had been diagnosed to have congenital iris and choroidal colobomas in both eyes at the age of four years. When examined, BCVA was 20/600 N36 in RE and 20/120 N6 in LE. Slit-lamp biomicroscopy showed bilateral inferonasal iris and lens colobomas. Fundus evaluation of RE revealed a choroidal coloboma involving the disc and macula with a rhegmatogenous retinal detachment sparing the coloboma. LE showed a choroidal coloboma involving the disc and extending up to the macula with subretinal hemorrhage and a pigmented subfoveal lesion. FFA [Fig. 3] and OCT showed a subfoveal CNV. She underwent reduced fluence (25 J) PDT with verteporfin using a spot of 1.5 mm followed 48 h later by an intravitreal injection of 0.05 ml bevacizumab (1.25 mg). She subsequently underwent scleral buckling in RE followed by lensectomy, vitrectomy, endolaser and silicone oil injection for recurrent retinal detachment. When last seen 11 months after treatment, BCVA was 20/80 N6 in LE with a regressed CNV [Fig. 4].

### Case 3

**Figure 5:**
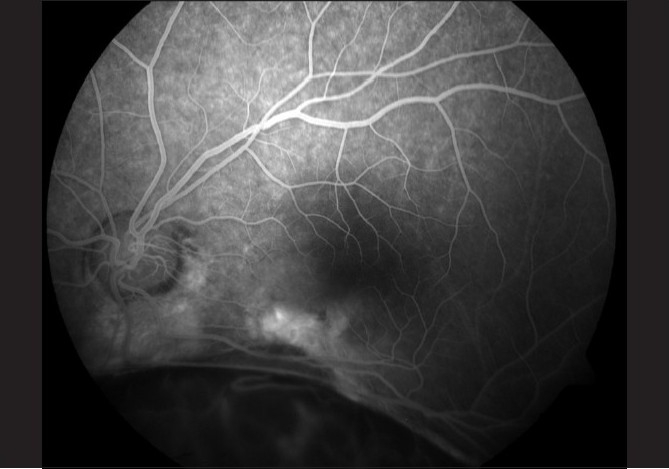
Pretreatment FFA of Case 3 showing a classic CNV at the coloboma margin

**Figure 6:**
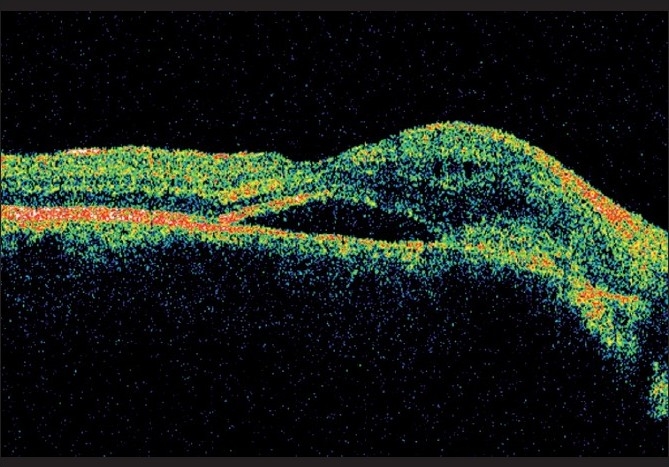
Pretreatment OCT of Case 3 showing subretinal fluid under the fovea and CNV at the coloboma edge

**Figure 7:**
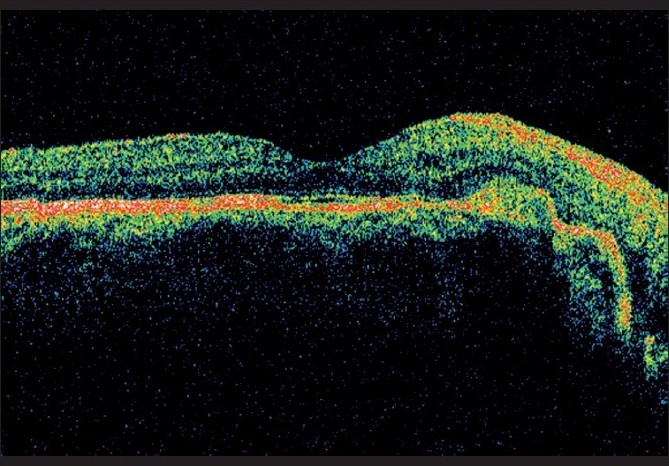
Post-treatment OCT of Case 3 showing resolution of subretinal fluid after PDT and bevacizumab

A 35-year-old male presented with metamorphopsia in LE for two weeks. The RE had poor vision since childhood. BCVA in RE was 20/2000 and in LE 20/32 N8. Anterior segment and intraocular pressures were unremarkable. Fundus evaluation of the RE revealed a large choroidal coloboma involving the disc and macula. The left fundus showed a normal disc and choroidal coloboma sparing the macula. There was a grayish lesion adjacent to the upper border of the coloboma with a streak of subretinal hemorrhage and subretinal fluid extending under the fovea. FFA showed a juxtafoveal classic CNV [Fig. 5] and OCT showed the presence of associated subfoveal fluid [Fig. 6]. After a detailed discussion with the patient regarding treatment options including laser photocoagulation, PDT and anti-vascular endothelial growth factor (VEGF) monotherapy, he underwent reduced fluence PDT with verteporfin followed 48 h later by an intravitreal injection of 0.05 ml bevacizumab (1.25 mg). Five months post treatment, BCVA was 20/20 N6 in LE with regressed CNV and absence of subretinal fluid on OCT [Fig. 7].

## Discussion

CNV occurrence in choroidal colobomas is believed to be due to preexisting anomalies at the border of the coloboma where there is thickened and folded Retinal pigment epithelium (RPE), retina is atrophic and the barrier function of Bruch’s membrane is disrupted. CNV though rare, assumes importance when the good eye of the patient is involved. Management of CNV in these eyes does not differ from that of CNV due to other causes. Laser photocoagulation has the disadvantage of causing significant scarring and hence a disturbing scotoma. This is however, a cheaper and viable option where the lesion is either extra- or juxtafoveal as in Case 3. Similar is the case of transpupillary thermotherapy (TTT) where treatment effects are unpredictable. PDT with verteporfin has been shown to be effective in reducing the risk of moderate to severe visual loss in patients with age-related macular degeneration (AMD)-related CNV[1] and has also been described in non-AMD CNV. Significant visual loss may still occur due to cumulative damage to the retina and inner choroid. Intravitreal anti-VEGF agents have become the first-line therapy for AMD- and non AMD-related CNV[2] though the treatment involves monthly evaluation and sometimes indefinite numbers of injections. The need to reduce the number of treatment sessions has prompted the use of PDT with anti-VEGF agents and this combination has been reported to be effective. Reduced fluence PDT has shown potential to minimize RPE destruction.

Case 1 was treated with standard fluence PDT and showed regression with RPE atrophy corresponding to the spot. In Cases 2 and 3, reduced fluence PDT combined with intravitreal bevacizumab achieved good results though anti-VEGF monotherapy was also an option that was taken into consideration. We have a fairly good follow-up in Cases 1 and 2, and even in Case 3 the fact that the patient was stable for five months post treatment speaks for the efficacy of the same.

Though retinochoroidal colobomas have been well-documented and researched, literature is scarce on the incidence and management of CNV, with most articles being case reports.[3–5]

Laser photocoagulation,[6,7] TTT,[8] PDT[9] and surgery,[10] have been reported. Our series of three cases of PDT for CNV associated with coloboma of the choroid is the largest in the literature, and the first where combination therapy with PDT and bevacizumab has been used. Though we have other cases where anti-VEGF monotherapy has been used, the follow-up in such cases is as yet not long enough for us to draw a meaningful comparison of the relative efficacy of each treatment modality. What is encouraging is that in all three of our cases reported here, a single treatment session appeared to achieve the desired results, suggesting that CNV in colobomatous eye may not be the result of an ongoing angiogenetic process, but a onetime event.
